# Asymmetry in the retention of content and surface linguistic information during reading in L1 and L2

**DOI:** 10.3389/fpsyg.2025.1610120

**Published:** 2025-07-08

**Authors:** Denisa Bordag, Andreas Opitz, Hans-Georg Berulava

**Affiliations:** ^1^Herder Institute, Leipzig University, Leipzig, Germany; ^2^Haifa University, Haifa, Israel

**Keywords:** memory, reading, eye tracking, mental text model, content information, surface linguistic information, native-non-native differences

## Abstract

This eye-tracking study investigates how native (L1) and non-native (L2) German speakers retain content and surface linguistic information during reading, drawing on the Construction-Integration Model of text comprehension. Participants read narrative texts, followed by picture and sentence reading tasks designed to assess memory for content and surface linguistic forms (e.g., grammatical voice, attribute position). Results reveal an asymmetric retention pattern: L1 readers demonstrated stronger retention of content information, indicated by longer fixation times on semantically incongruent pictures and sentences. In contrast, L2 readers showed enhanced retention of surface linguistic forms, evidenced by extended fixations on sentences with altered surface structures. These findings align with the Shallow Structure Hypothesis and the Declarative/Procedural Model, suggesting that L2 readers rely more heavily on declarative memory for surface forms due to less automatized syntactic processing. By directly comparing L1 and L2 retention patterns, this study provides novel insights into the mental representation of text in L2 readers, highlighting an increased retention of surface information that is accompanied by reduced content retention.

## 1 Introduction

When confronted with a statement like “The language we use affects how we think and act,” our attention typically turns to differences between the world languages and how they might shape our cognition (Sapir-Whorf hypothesis / linguistic relativity hypothesis, Bohnemeyer, 2020; Lucy, 1992; Whorf, 1956; Wolff and Holmes, 2011). However, growing evidence suggests that we could also refer to differences that emerge from using one’s native (L1) or non-native (L2) language. Such differences might be of a general nature that is independent both from particular linguistic differences between individual languages and from potential deficits in mastering the grammar and vocabulary of a given L2.

As an example, some studies on decision-making behavior suggest that we evaluate described events and dilemmas differently, depending on whether they are presented to us in our L1 or L2 (so-called “Foreign Language Effect” hypothesis, Keysar et al., 2012). The results indicate that the use of an L2 might promote a more deliberate and analytical mode of thinking, as the decisions made in response to situations described in an L2 are often more rational and less emotionally influenced.

Less intense emotional responses in L2 are suggested also by literature directly addressing emotional depth. According to Dewaele (2004), emotional terms and concepts in one’s native language evoke deeper emotional reactions because they are closely tied to cultural and personal background. Research on embodied cognition (Pavlenko, 2012) and neurocognitive studies (Harris et al., 2006; Hsu et al., 2015) further suggest that emotional terms in an L2 are less strongly linked to emotional centers in the brain, such as the amygdala, leading to weaker emotional responses compared to the stronger activation observed when processing emotional terms in the L1.

In the current study, we focused on another cognitive dimension that can be affected depending on whether we use our L1 or L2, namely memory and in particular retention of information during reading. When we read, we construct a mental text model that represents our memory of what we have just read. In 1983, van Dijk and Kintsch proposed a model of text comprehension, which was further developed into the tripartite Construction-Integration Model (CI Model; van Dijk and Kintsch, 1983; Kintsch, 1988a,b, 2018) and which remains a framework of reference in contemporary cognitive and psycholinguistic research. The model conceptualizes text comprehension as a multi-level process. Its two primary levels target the representation of the text content. They are the *textbase level*, which represents the literal meaning of the text, including its semantic structure and propositions, and the *situation level*, which goes beyond the text itself by integrating prior knowledge and contextual understanding to construct a richer mental representation of the information. In addition, there is a third level that has been referred to as the *linguistic representation level* (Wharton and Kintsch, 1991), *verbal/linguistic level* (Kintsch and Welsch, 2013), or *surface structure* or *surface level of representation* (both Kintsch et al., 1990). This level encompasses surface linguistic information—that is, the literal representation of the text, including its exact wording, syntactic structure, and grammatical form—which serves as a foundation for constructing the textbase. As Kintsch and Welsch (p. 3) acknowledge, this level has often been neglected in the model, despite being addressed in some publications (e.g., Kintsch et al., 1990). Its mental representation is assumed to be short-lived and to decay rapidly. Consequently, it is not typically preserved in long-term memory, which primarily encodes the semantic and conceptual content represented at the two higher levels of the model (Anderson, 1974; Gernsbacher, 1985; Just and Carpenter, 1992; Kintsch and van Dijk, 1978; Lombardi and Potter, 1992; Rummer and Engelkamp, 2001; Sachs, 1967, 1974; Garnham and Oakhill, 1996; Johnson-Laird, 1977).

The assumption that surface-level linguistic information decays rapidly originated in experimental work conducted during the 1960s and 1970s. In these studies, participants listened to or read isolated sentences or brief texts and were later presented with a sentence and asked to decide whether it was identical to one they had encountered previously. Successful recognition of the original sentence wording was interpreted as evidence for surface form retention (also called verbatim memory). The structures or alternations examined to test the retention of surface linguistic information varied in type and in processing complexity: some involved grammatical manipulations, others involved changes in information structure, and some consisted of purely formal modifications. What these variations had in common was that they differed in their surface forms but did not alter the sentence’s factual meaning. Accordingly, they were assumed not to affect the construction of propositional content at the textbase level, thereby enabling researchers to isolate the contribution of surface-level retention. Among the investigated structures were, for example, voice alternations (e.g., active vs. passive; Sachs, 1967, 1974; Anderson, 1974), shifts in the position of appositions (Sachs, 1967), alternations in double object constructions (Soli and Balch, 1976), synonym substitutions (Sachs, 1974), and purely formal changes such as word order shifts (e.g., “A wealthy manufacturer, Matthew Bolton, sought out the young inventor.” vs. “A wealthy manufacturer, Matthew Bolton, sought the young inventor out.”; Sachs, 1974) (see Opitz et al., 2024 for more detail).

The conclusions about the fast decay of surface linguistic information, however, have not gone unchallenged. In 2010, Gurevich et al. (2010) and colleagues carefully reviewed the previous literature on the topic and concluded that memory for linguistic surface information does not seem to be so absent in naturalistic contexts as claimed. For example, they refer to a study by Kintsch and Bates (1977), which demonstrated that participants were significantly better at recognizing the exact wording of sentences they had heard in a regular university lecture than at recognizing paraphrased versions of those sentences. In their own experiments, Gurevich and colleagues further showed that memory for exact wording in naturalistic settings persists longer than had been assumed (cf. Gibbs, 1981; Kintsch and Bates, 1977).

Moreover, recent research has indicated that retention of surface linguistic information may be more prominent in L2 than in L1, pointing toward differences between the L1 and L2 mental text models. For example, in Bordag et al. (2021), L2 readers retained information on whether sentences had appeared in a text in the active or passive voice, while no retention effects could be observed in L1. Similar results have been reported also in studies focusing on single sentences. Sampaio and Konopka (2013) showed an L2 advantage for the retention of the linguistic surface form when words were substituted with their synonyms (e.g., “The bullet struck/hit the bulls’ eye”). Bordag and Opitz (2025) replicated the L2 retention advantage for surface linguistic information for active and passive voice at the sentence level, and added further evidence for L2 verbatim retention of various verbal tense forms (preterit vs. perfect) referring to the same time (past) in German. Opitz et al. (2024) further showed that at the sentence level, L1 German readers are also able to retain surface-level linguistic information (active and passive voice and adverb usual position vs. adverb fronting), though the retention effects were sometimes smaller than in German L2.

At the same time, some of the sentence-based studies indicated that better retention of surface linguistic information in L2 might be accompanied by worse retention of conceptual information. Bordag and Opitz (2025) observed that while L1 participants could retain information about grammatical number in specific contexts (singular vs. plural) or about tense (present vs. past), L2 participants did not retain this information unless it was expressed by robustly different forms (e.g., tense differences between analytical and synthetic forms, but not between synthetic forms only). However, this study addressed only grammatically instantiated conceptual information (like tense information), not other types of content information.

Interestingly, as pointed out by Opitz et al. (2024) and Bordag and Opitz (2025), the overrepresentation of linguistic surface-level information in L2 mental text models aligns with key assumptions of L2-processing theories—particularly the Shallow Structure Hypothesis (SSH) proposed by Clahsen and Felser (2006, 2018). According to SSH, L2 learners possess the same processing architecture and cognitive mechanisms as native speakers, yet they have “problems building or manipulating abstract syntactic representations in real time” (Clahsen and Felser, 2018, p. 694). Consequently, they tend to underuse morphosyntactic information in online processing and rely more on “semantic, pragmatic, probabilistic, or surface-level information” (Clahsen and Felser, 2018, p. 694).

In this context, surface-level information refers to the literal form of the sentence—such as exact wordings, word order, and surface morphosyntactic forms— distinct from the grammatical parsing or interpretation of these forms during online processing: While L2 readers may struggle to parse and interpret complex grammatical structures on the fly, they might still be capable of retaining and even relying on these forms as unanalyzed chunks in memory. This aligns also with the assumptions of Ullman’s Declarative/Procedural (DP) Model (Ullman, 2001, 2004, 2016), which provides a neurocognitive explanation for the differing reliance on memory systems in L1 and L2 language use. The model posits that while native speakers typically acquire and process grammar using procedural memory, adult L2 learners are less efficient in accessing this system, particularly for morphosyntactic processing. Instead, they compensate by relying more heavily on declarative memory, which supports the retention and retrieval of factual knowledge, including vocabulary and surface forms of language.

It is noteworthy to mention that usage-based approaches to language acquisition suggest that accumulation of surface-form exemplars in memory might directly contribute to grammatical learning. These approaches, rooted in cognitive linguistics, emphasize that grammatical knowledge emerges from language use and is grounded in experience. The foundation of grammar is thus a rich inventory of memorized chunks of language—surface-level form-meaning pairings that are stored in memory and reused across contexts (Bybee, 1985, 2010; Ellis, 1996; Goldberg, 2006; Langacker, 1988; Tomasello, 2003). Over time, these stored exemplars support the abstraction of grammatical regularities and the formation of schematic constructions, enabling the gradual emergence of mental grammar.

Overall, recent research on language comprehension and mental text models, L2 processing approaches such as the SSH and the DP Model, as well as usage-based theories of language acquisition, suggest that the presence of surface linguistic information in L2 mental text representations may not be merely incidental but may reflect adaptive cognitive strategies shaped by underlying processing constraints and contributing to grammar acquisition.

Despite these potential advantages, it remains an open question whether the overrepresentation of surface linguistic information by L2 readers (compared to L1 readers) is accompanied by an ability to retain content-level information in an extent comparable to that of L1 readers, or whether the allocation of cognitive resources to surface-form retention is accompanied by poorer memory for propositional content. This constitutes the central research question addressed in the present study.

## 2 The present study

In the present study, we investigate memory for both *surface linguistic information* and *content information* during text reading in L1 and L2 German. To our knowledge, such a direct comparison has not yet been conducted. Specifically, we aim to explore whether these two types of information are represented to different extents in L1 and L2 mental text models. We hypothesize that advanced L2 readers (B2/C1 level) will outperform L1 readers in retention of surface-level linguistic information while exhibiting weaker memory for content information — consistent with findings on conceptual feature retention reported in Bordag and Opitz (2025). These research questions are not only of theoretical significance as outlined in the Introduction, but also have practical implications. Understanding L1-L2 differences in text models could shed light on why L1 and L2 reading experiences differ and why studying from texts in a non-native language may pose additional challenges, even when L2 proficiency is sufficient for achieving full text comprehension.

In order to address these questions, we employed an identical/changed eye-tracking paradigm that builds on a design originally developed for research on the retention of surface-level linguistic information (Bordag et al., 2021; Bordag and Opitz, 2025; Opitz et al., 2024), adapted here to also include a task focusing on content information retention. The paradigm’s core idea is that readers can detect deviations from previously presented information only if they have stored the original information in memory. To test this, information is presented twice: first in a text and later in a follow-up task. In the identical condition, the information presented in the text and in the follow-up task is the same; in the altered condition, the information in the follow-up task differs from that presented in the text. Longer fixation times in the altered condition compared to the identical condition during the follow-up task indicate that participants detected the change, i.e., they retained the original information as it occurred in the text and experienced surprisal or processing difficulty during the follow-up task, when encountering the deviation from the stored representation.

*Retention of surface linguistic information* was assessed using a sentence-reading follow-up task. In this task, the sentences either matched or diverged in surface linguistic form from those presented in the text, in accordance with the experimental rationale described above. We examined two grammatical properties, voice (i.e., the active/passive alternation) and attribution (i.e., left adjectival attribute / right relative clause attribute). Both of these alternations affect the surface realization of the sentence, but do not change its propositional meaning. We decided to use the voice alternation because previous text studies (Bordag et al., 2021) showed that, while L2 participants retain information about whether a sentence was presented in a passive or in an active voice, L1 participants typically do not. Thus, we expected to replicate this finding and to validate that the paradigm also works in the adapted version. The attribution alternation was selected because we wanted to test an additional alternation that we had not previously investigated to ensure that the retention effects are not specific to particular realizations (such as active and passive constructions). Moreover, this alternation is formally very saliently marked and involves crossing a clause boundary. We wanted to test whether an alternation with different properties could affect the surface linguistic form retention differently.

Voice alternation in German (and also in Slavic languages which were the L1 of the participants) involves a morphosyntactic shift between active and passive and is similar to the voice alternation in English: In the active voice, the agent is the subject of the sentence, and the patient is an object in the accusative case. In the passive, the patient becomes the subject, while the agent can appear in a prepositional phrase (e.g., *Die Umzugshelfer trugen die Möbel.* vs. *Die Möbel wurden von den Umzugshelfern getragen.* — “The movers carried the furniture.” vs. “The furniture was carried by the movers.”). Passive formation is analytical, requiring an auxiliary and a participle. In contrast to English and the Slavic languages, the non-finite participle form is in the sentence-final position in German.

In the attribute alternation, the attribute either precedes the noun as an adjectival attribute or follows it in the form of a relative clause, e.g., *Ein besonders dickes Schwein / Ein Schwein, das besonders dick war, lag schlafend in der Ecke.* (“A particularly fat pig / A pig that was particularly fat was lying asleep in the corner.”). The structure of both types of attributes is similar in German and in the Slavic languages, except that in German relative clauses the verb is at the end of the sentence.

*Retention of content information* was tested in two ways. Both were based on the inclusion of easily visualizable scenes within short text stories. In the first, non-verbal viewing task that followed the text, these critical scenes were depicted as pictures. The scenes were either presented congruently with the text or differed from the text scene in one element. For example, if the text mentioned the sentence: *Ein kleiner Junge mit Brille malte mit Kreide einen Dinosaurier auf den Asphalt.* (“A little boy with glasses was drawing a dinosaur on the concrete using chalk.”), the picture depicted either the very same scene, or a scene in which the same boy was drawing a car (or vice versa in another text-picture version). The expectation was that if readers remember the information about what the boy was drawing, they would notice the deviation from the text when viewing the picture. Participants’ sensitivity to the information mismatch in the incongruent condition would manifest itself in longer viewing times at the critical region of the picture compared to the condition in which the picture scene was text-congruent.

**Figure 1:**
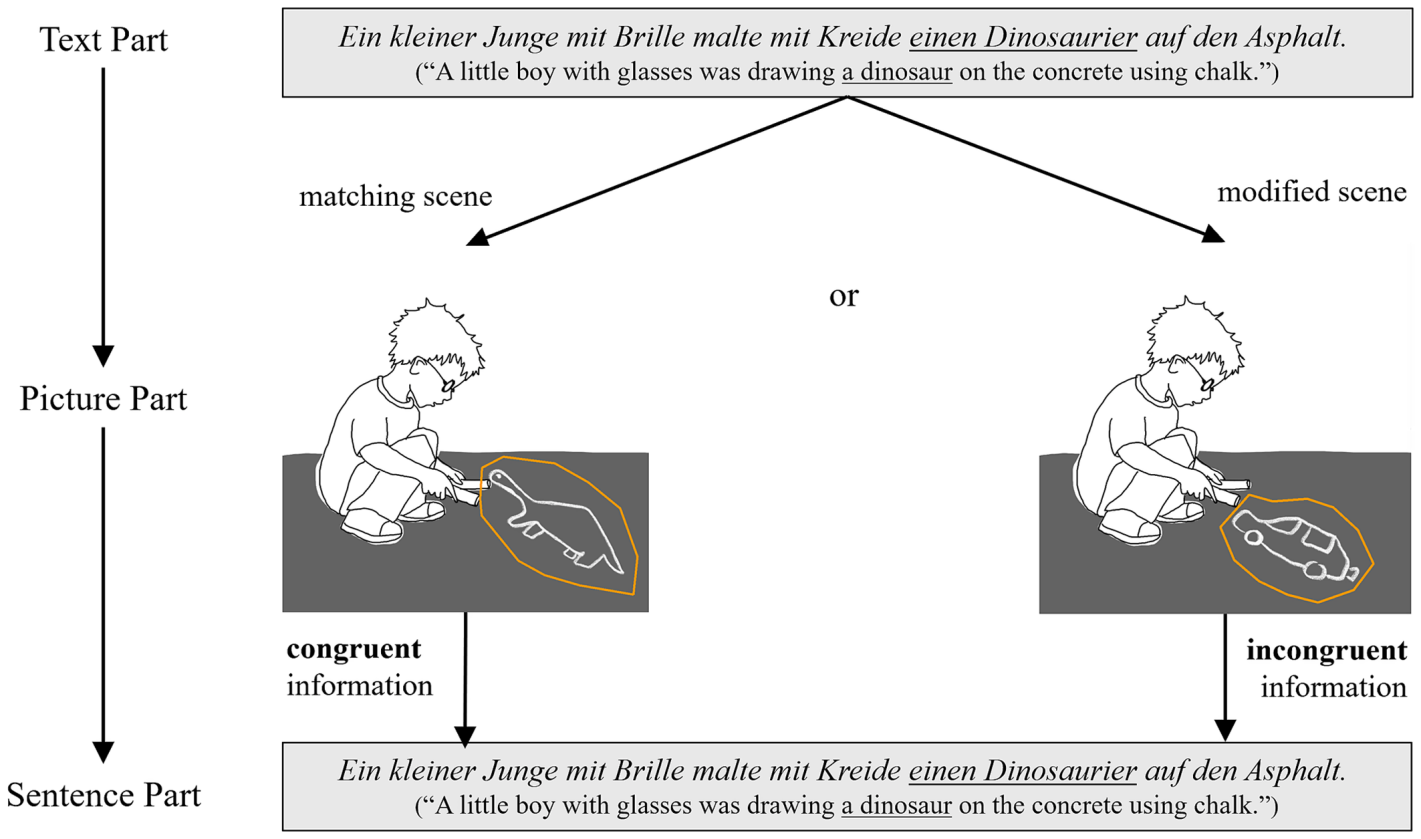
Example of one critical content sentence across the experimental tasks and conditions. For illustration, regions of interest (containing the manipulated object) are depicted here (but they were not visible for participants).

In the second task, the verbal follow-up task, the same sentences that described the critical scenes in the text were presented individually, without additional context. Although the sentences in the text and the follow-up task were formally identical, they differed in terms of content congruency. This was achieved via the aforementioned picture viewing task, which took place between reading the text and completing the second follow-up task (i.e., reading the sentences). In the picture task, half of the scenes depicted were text-scene congruent and the other half were incongruent. Thus, when reading the sentence in the second follow-up task, participants were either presented with a content-congruent information for the third time (e.g., a boy drawing a dinosaur in the text, in the picture, and in the individual sentence) or they were presented with incongruent content information throughout the experiment via the intervening picture task (e.g., a boy drawing a dinosaur in the text, a boy drawing a car in the picture, and a boy drawing a dinosaur again in the text), see Figure 1. If participants had retained previous content information from the text and the pictures, we expected that in the congruent condition, the total fixation time on the sentence would be shorter than in the incongruent condition, since participants would be processing the same information for the third time. In the incongruent condition, however, they should spend more time on reading the sentence, since from their perspective it was not clear which content information it would refer to (the text or the picture). However, if participants had only a vague memory of which information preceded, they should show no substantial difference in processing the sentence in both conditions.

We also added a number alternation (i.e., singular/plural) to the sentence task, similar to the surface linguistic alternation. In contrast to the previous two alternations, which affected the formal realization of the sentences but not their propositional meaning, the alternation of number information involves both formal (inflectional suffix in plural, agreement changes on the article and the verb) and imageable conceptual changes (one vs. several) and thus also modifies the meaning of the sentence: (e.g., *Das Umzugsauto stand / Die Umzugsautos standen bereits vor ihrem Haus.* — “The moving van was already standing / The moving vans were already standing in front of their house”).

In our previous study (Bordag and Opitz, 2025), only L1 participants were sensitive to the number alternation. For the L2 participants, whose retention was driven more by the formal aspects, the inflectional endings were probably not salient enough. The retention of the number information probably failed due to difficulties in processing inflectional morphology (see SSH, Clahsen and Felser, 2006, 2018): the not (fully) processed information could not be retained. However, additional analyses of the 2025 data revealed that L2 participants were able to retain (and thus also process) the number information expressed by an inflectional suffix, but only when the manipulated NPs appeared in syntactically prominent subject position. Thus, in the current study, we decided to include the number alternation again, but this time all number-manipulated NPs appeared in the subject position. We wanted to see whether syntactic prominence would contribute to the retention of number information in L2.

## 3 Methods

### 3.1 Participants

Sixty-four native German speakers [mean_age_ = 25.9 years; SD = 5.0 years; range = 19–39 years, 43 female, 20 male, 1 diverse] and 64 L2 German speakers with a Slavic L1 (43 Czech, 3 Polish, 14 Russian, 1 Slovak, 3 Ukrainian) [mean_age_ = 23.8 years; SD = 4.9 years; range = 18–45 years, 52 female, 12 male] participated in the experiment. The L2 participants’ knowledge of German was pre-advanced to advanced, ranging from the proficiency levels of B2 to C1 according to the Common European Framework of Languages (CEFR). Proficiency levels were assessed using a combination of three measures: a self-reported linguistic background questionnaire (the questionnaire can be found in the online repository https://osf.io/b6dr4/), the score from participants’ most recent official test and the result from the DIALANG placement subtest (Alderson, 2005). Only participants who reached levels B2 and C1 in all three assessments were tested in the experiment (most participants did not achieve identical scores in all three assessments, but did achieve both B2 and C1 depending on the test).

All participants had normal or corrected-to-normal vision and reported neither reading nor cognitive impairments. They signed a consent form before the experiment and received monetary compensation.

### 3.2 Materials

The experiment consisted of four main trials, each with the same three components: (1) a German-language text to read, (2) a set of pictures to view, and (3) individual sentences to read. The initial text in each trial served as the basis, with information in subsequent picture tasks and sentence tasks either aligning with it or diverging from it.

The four texts were designed in two versions, V1 and V2, which differed with respect to manipulations targeting the research questions. Each participant was presented with only one version. The pictures consisted of a subset of pictures that either exactly depicted scenes from the text (congruent condition) or deviated from them (incongruent condition) and of a subset of filler pictures completely unrelated to the text. Similarly, to probe memory for surface linguistic information, a subset of critical sentences was either identical to the sentences that appeared in the text (identical condition), or they deviated from them with respect to targeted properties (changed condition). Another subset of critical sentences was related to the previously viewed pictures (to probe retention of content information).

All materials, i.e., 8 texts, 96 pictures and 224 sentences, were distributed across four experimental lists according to a Latin square design so that they were cross-balanced with respect to all manipulations, i.e., by presenting identical or manipulated versions of pictures and sentences in comparison to the text that was read initially (in one of the two versions). The order of the four main trials was randomized, as was the order of presentation of the pictures and sentences within each main trial.

#### 3.2.1 Texts

The four texts, titled *Auf dem Land* (*In the Countryside*), *Die Demonstration* (*The Demonstration*), *Im Zoo* (*At the Zoo*) and *Der Unglückstag* (*The Unlucky Day*), were between 802 and 930 words long. They were written in a narrative style and contained a number of vivid pictorial descriptions of the scenes included. A pre-test was conducted to ensure that the texts were suitable for B2 readers at both the lexical and grammatical level. In the pre-test, 12 non-native speakers of German with a Slavic L1 read all four texts as well as two filler texts, and highlighted words or expressions they were not familiar with. Problematic text passages of the test material were then simplified based on the responses.

Each text existed in two versions that differed in the realization of 20 critical sentences each. Four sentences were manipulated with respect to their voice, four with respect to the attribution (thus there were eight critical sentences in each text for the manipulation of the surface linguistic form information). Eight further critical sentences in each text differed in their realization with respect to their content (for the content manipulation). Additionally, in each text there were four sentences to test number information. Each participant was presented with only one version of the text in which these critical sentences always appeared either in the order ABAB or BABA with respect to their two possible realizations (e.g., for voice: A – active, B – passive).

The voice manipulation was performed on four sentences, such that in one version of the text two critical sentences were in the active and two in the passive and in the second version the voice realization in the four critical sentences was reversed, e.g., *Ein junger Mann hob die Tasche der Dame auf* (*A young man picked up the lady’s bag*) vs. *Die Tasche der Dame wurde von einem jungen Mann aufgehoben* (*The lady’s bag was picked up by a young man*). The critical sentence never included pronouns, nor particle or prefix verbs. The patient was always realized using the accusative case. A maximum of one complement in addition to the subject and objects was used. The regions of interest (ROI) in the sentence reading follow-up task included the complete critical sentence, as voice manipulations involve changes affecting the whole sentence.

The manipulation of the attribution was performed on four sentences: The attribute preceded the noun phrase in the form of an adjective or participial phrase in two instances, and followed it as a relative clause in two instances, e.g., *Auf dem Schoß der Frau saß ein laut schreiendes Baby (A loudly crying baby was sitting on the woman’s lap)* vs. *Auf dem Schoß der Frau saß ein Baby, das laut schrie (A baby was sitting on the woman’s lap, who was crying loudly)*. Attributes preceding the noun phrase were at most two words long and, in the case of participial phrases, never included a prepositional phrase. The ROIs in the sentence reading follow-up task included the noun (with its article) and the whole attribute (as underlined).

The number manipulation was performed on four sentences, so that the critical noun phrase was in the singular or plural in two instances each, with further syntactic elements, such as verbs, exhibiting agreement, e.g., *Der Getränkeautomat war leer* (*The beverage dispenser was empty*) vs. *Die Getränkeautomaten waren leer* (*The beverage dispensers were empty*). The critical noun phrase was always the subject of the sentence and appeared with either a definite article or a demonstrative. Only nouns with a distinct plural form including a suffix were included, but never feminine^1^ or abstract nouns. The singular-plural difference had to be clearly salient and imageable in the given context. Half of the respective nouns denoted animate objects, the other half inanimate objects. The agreeing verb was placed as closely as possible to the subject, and prefix or particle verbs were avoided. The ROIs in the sentence reading follow-up task included the critical noun phrase plus one following word (spill over).

The eight sentences that were manipulated with respect to their content, as well as the filler content sentences, were written in such a way that they could be depicted visually later in the experiment. They did not include reference to any colors, as they were to be illustrated only in black and white, and a maximum of three referents were included in order to avoid overly complex illustrations. The sentences were also formulated in such a way that they could be presented in a manipulated version as a picture that differed in only one element. The noun representing the changing element always had the syntactic function of either a subject or an object (i.e., no adjuncts) for increased salience in perception, e.g., *Ein kleiner Junge mit Brille malte mit Kreide ein Auto / einen Dinosaurier auf den Asphalt.* (*A little boy with glasses was drawing a car / a dinosaur with chalk on the asphalt.*). Moreover, half of the sentences contained an action verb, while the other half contained a stative verb. Only the noun phrases that were the subject of the content manipulation were designated as ROIs.

All critical sentences complied with the following criteria: Manipulations at the end of sentences were avoided to prevent distorted measurements due to wrap-up effects and peripheral vision. Lexical words were never repeated across critical sentences. Critical sentences manipulating linguistic properties were not allowed to follow each other; the same criterion applied to critical content sentences. However, both types of sentences could appear consecutively in the text. There were no subject-object inversions at the beginning of the critical sentences. Critical content sentences referred to facts that could not be derived from general world knowledge.

#### 3.2.2 Pictures

After reading a text, participants were presented with a block of pictures. There were 24 pictures per each of the four main trials which were created manually in a digital format by a graphic designer. They were simple illustrations in a square format of 2048×2048 pixels, in the form of black and white drawings with two types of grey shading.

For each trial/text, eight pictures were used to implement the content manipulation. Four pictures depicted exactly the proposition of the four critical content sentences in the given text (congruent condition). The other four critical pictures involved a deviation from the proposition compared to the four other critical content sentences in the text (incongruent condition), see Figure 1 for an example. In addition, participants were presented with eight pictures serving as fillers. Four of them depicted non-critical sentences from the text in an identical or modified way, and four were completely unrelated pictures which did not resemble any of the texts’ content.

The ROIs in the pictures were manually defined using a visual markup interface and covered the critical element of the picture that was manipulated (i.e., either matched or mismatched the critical referent from the corresponding sentence in the text).

Note that for the critical sentences there were always two text versions and two picture versions that were fully cross-balanced and distributed over four experimental lists according to a Latin square design.

#### 3.2.3 Sentences

The picture presentation was followed by a block of sentences. There was a total of 36 sentences per main trial. Eight sentences were designed to create the surface linguistic manipulations (voice and attribution) and four to create the number manipulation. They were either identical with one of the critical sentences in the text (identical condition) or they were altered with respect to one of the three critical variables (changed condition). Thus, as an example, one active and one passive sentence remained identical, as in the text, while one passive sentence was changed to active and one active sentence was changed to passive.

Eight further sentences tested the influence of the corresponding picture on the retention of the information presented in the critical content sentences in the texts. They were all identical with the corresponding sentences in the text. For half of the sentences, there was a complete information/content match also with the corresponding picture (the congruent condition). For the other half of the sentences, the corresponding picture involved a deviation from the target sentence and thus also from the corresponding sentence in the text (the incongruent condition).

In addition, there were 16 filler sentences. Eight of them appeared identically in the text, of which two had been depicted in the picture block accordingly and two in a modified manner. Four filler sentences described previously viewed pictures unrelated to the text, and four additional filler sentences were completely unrelated to both the text and to the pictures. The purpose of the filler sentences (and pictures) was to prevent participants from detecting regularities in the experimental design.

**Figure 2:**
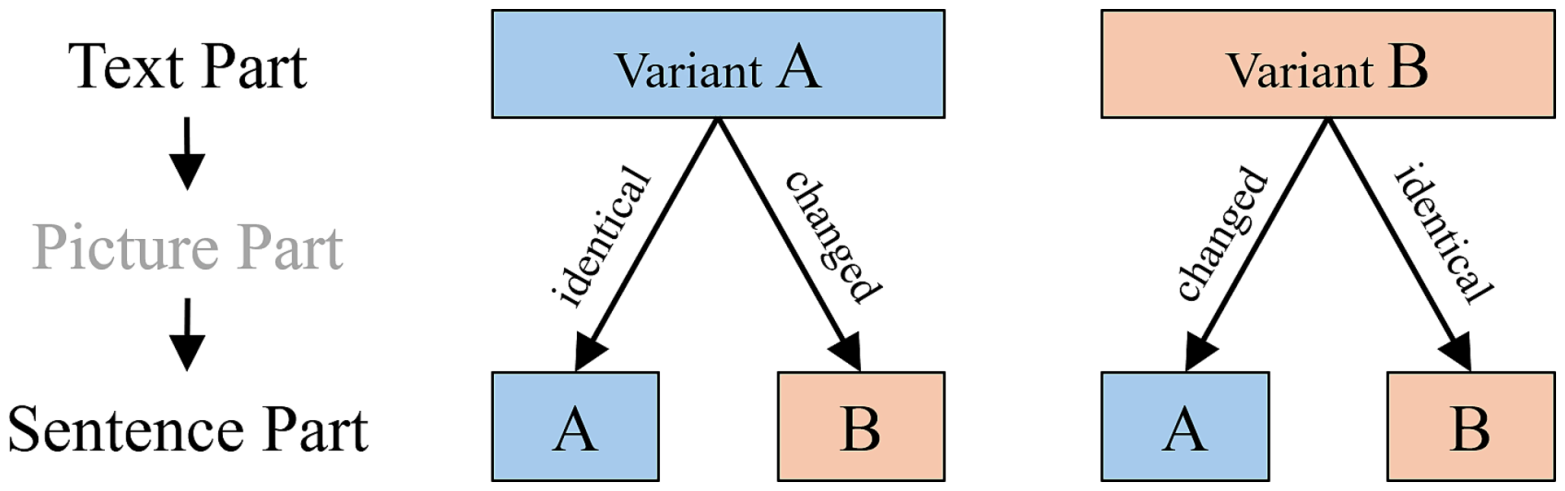
Scheme of the experimental manipulation of surface linguistic information.

Importantly, in the identical/changed paradigm, both variants of an alternating sentence pair (e.g., active or passive) are completely counterbalanced with respect to which member of the pair appears in the first and second presentation and in a changed/identical condition. This results in four different combinations (see Figure 2).

All materials (including texts, pictures, and sentences) are provided in the online data repository accompanying this paper on OSF: https://osf.io/b6dr4/.

### 3.3 Apparatus and procedure

The experiment was programmed using the EyeLink Experiment Builder software (version 2.4.193, SR Research, 2023). It was run on an Asus ROG Zephyrus S17 laptop connected to a Lenovo 480 laptop via a LAN cable. An EyeLink Portable Duo eye tracker was installed on a laptop mount, and participants placed their chins on the EyeLink Table Clamp Chin Cup to maintain a stable head position. Reading was recorded binocularly at a sampling rate of 500 Hz. The screen resolution was 1920×1080 pixels. The text was displayed in a left-aligned 16-point black sans-serif font (‘Calibri’) on a light grey background. Each text was divided into sections of between 104 and 177 words which were presented on six individual screens, with critical sentences never appearing directly at the beginning or end of a screen if possible. Individual sentences were presented in the same way, but vertically centered. They were left-aligned with a 250-pixel margin. The square-shaped pictures were displayed in the center of the screen with a height of 1,000 pixels, a width of 1,000 pixels, and balanced top and bottom margins (40 pixels each).

Participants were tested individually in a quiet, dimly lit room while seated comfortably in front of the presentation laptop at a distance of approximately 60 cm from the eye tracker. After filling in the linguistic background questionnaire (including information on participants’ native language and foreign languages learned, such as language proficiency level and contexts of acquisition) and, in case of the L2 participants, completing the DIALANG vocabulary placement test (Alderson, 2005), participants were instructed regarding the procedure of the experiment.

The experiment started with a practice trial that was designed analogously to the main trials but was reduced in scope by 50%. After familiarizing themselves with the experimental setting and procedures, participants completed the four main trials comprising a text, picture and sentence block each. Participants had short breaks between the main trials of the experiment. The overall duration was approximately 70 min for L1 participants and 100 min for L2 participants.

The eye tracker was calibrated using EyeLink’s 13-point-calibration before the presentation of each text or set of individual sentences. Each section of a reading text and each individual sentence was preceded by an empty screen with a fixation point in the top left corner for texts and in the center left for individual sentences. Once a participant fixated on it, the experimenter displayed the respective text part or a sentence. After the participants completed reading the text or a sentence or viewing a picture, they pressed a designated button on the response box to proceed.

During the picture block, participants were asked to answer questions about four of the filler pictures in order to maintain their attention. The binary questions were presented on the screen and related to an aspect of the picture viewed immediately before, e.g., *Trägt der Polizist eine Brille?* (*Is the policeman wearing glasses?*). Participants responded by pressing a designated button on the MilliKey Response box connected to the presentation laptop. Later analyses showed that there was no statistical difference in the response accuracy of the L1 (94.6%) and L2 (93.1%) participants on this task.

In addition, after finishing each of the four main trials, participants completed a pen-and-paper questionnaire containing binary questions about whether specific sentences had been read before, i.e., *Kommt dieser Satz im Text oder in den einzelnen Sätzen vor?* (*Does this sentence appear in the text or among the individual sentences?*), and about the content of the text, e.g., *Gibt es im Dorf eine Kirche?* (*Is there a church in the village?*). The aim of the task was to motivate participants to read the text and the sentences thoroughly and to carefully view the pictures. The results showed a very high rate of correct answers in general (90.1%). The proportion of correct answers to questions related to the sentence was equally high for both populations (L1: 91.7%, L2: 91.6%). The text-related content questions were answered slightly better by the L1 participants (90.8%, L2: 86.1%). Taken together, the results of the additional question tasks suggest that the participants read the texts and sentences carefully and looked at the pictures attentively.

### 3.4 Data pre-processing and analysis

Prior to statistical analyses, all eye-tracking data was pre-processed using the software DataViewer (version 4.1.211; SR Research, 2019) to detect fixations and saccades using the software’s default settings. Additionally, the software’s automatic 4-stage fixation cleaning was performed with default settings for minimum and maximum fixation durations.^2^ Although data was recorded binocularly, gaze data was analyzed from the dominant eye for each participant.

In the following, the pre-processing steps and the analyses will be reported according to the order in which they appeared in the experiment for better understanding, i.e., first the analysis of the picture task, followed by the analyses of the content sentences, both of which address the content manipulation, and then the analyses of the sentences addressing the retention of surface linguistic information (voice and attribute), followed by the analyses of the number-manipulated sentences, which address both content and surface linguistic manipulations.

For analyses of the picture task, a critical ROI including the manipulated element was manually defined for each picture before the experiment was conducted (with an approximate margin of 10–20 Pixels, see examples in Figure 1). Note that all comparisons for the congruent versus incongruent meaning conditions were carried out within items (but between subjects): Each picture (e.g., depicting a boy drawing a dinosaur) contributed equally to the congruent and incongruent condition. Thus, although the critical ROIs in the pictures (the area of the manipulated object) were not identical across items, they were identical for each picture (within item) in the congruent and incongruent conditions.

For analyses of sentence trials, ROIs for each word were automatically defined with the software’s default settings for text with 30-pixel margins to the right and left of the text and with 60-pixel margins above and below the text. For the analyses reported below, ROIs were defined as follows: For both the content manipulation and the number manipulation, the critical noun phrase (i.e., the definite article and the noun) and the next following word (spill-over) were defined as the ROI. For the manipulation of the attribute position, the whole complex noun phrase was defined as the ROI: In the case of a left-attribute, this included (PP) + definite article + adjective + noun (e.g., *das sechs Jahre alte Mädchen* “the six-year-old girl”), in case of the right-attribute, this included the noun and the following relative clause: definite article + noun + relative pronouns + adjectival phrase + auxiliary (e.g., *das Mädchen, das sechs Jahre alt war* “the girl that was 6 years old”). Finally, for the manipulation of the grammatical voice (active versus passive), the whole clauses were defined as ROIs. This was done because comparisons between both voice alternations involved changes in word order throughout the clause, including the insertion of an auxiliary verb and a shift of the main verb (in participle form) to the last position of the sentence.

All experimental trials were further scanned manually, and drift correction was performed if necessary. For the analysis of the sentence task, the tool ‘Get Reading Measure’ provided by DataViewer was used to obtain reading time measures for the ROIs. The main reading measure analyzed in the present study was total fixation duration^3^. Total fixation duration refers to the sum of all fixations of a participant on a specific ROI in each trial and is equivalent to the cumulative amount of time that a person’s gaze is fixed on that ROI.

For all statistical analyses reported below, the software R was used (version 4.4.3; R Core Team, 2025). The data was analyzed with mixed-effects regression modeling using the R package *lme4* (version 1.1–33, Bates et al., 2015).

All models included fixed effects for all variables of interest and their interactions: Language (L1 vs. L2) and Condition. For Condition, factor levels coded the experimental manipulation, i.e., for the surface linguistic manipulation in the sentence task, factor levels were either ‘identical’ or ‘changed’ according to the repetition of the sentence. For the content manipulation in the picture task and the following sentence task, factor levels coded whether either a ‘congruent’ or ‘incongruent’ picture relative to the propositions presented in the text was shown. Beyond fixed effects and their interactions, the model structures included error terms for items and participants. Because models with the maximum structure for error terms (including random intercepts and random slopes for all predictor variables and their interactions as justified by the data structure) did not converge, we used the R package *buildmer* (version 2.9, Voeten, 2023) to identify the maximal structure for error terms that was still capable of converging. Starting with an empty random effects structure, terms were gradually added to the model until further additions prevented convergence. The order of adding terms was based on the Akaike information criterion (AIC). Final model structures are reported for each of the analyses below. The significance of fixed effects was evaluated using the R package *lmerTest* (version 3.1–3, Kuznetsova et al., 2017), and corresponding ANOVA (type III) tables with Satterthwaite approximation for degrees of freedom are reported below. All bivalent categorical predictor variables were effect-coded (i.e., as −0.5/+0.5).

The following OSF repository contains the datasets analyzed for this study, the R scripts to reproduce the statistical analyses, and all materials: https://osf.io/b6dr4/.

## 4 Results

### 4.1 Content information

Memory for content information during reading was probed in two tasks in the present study. The primary task was the *picture task*, in which participants saw a depicted scene that was either congruent with a scene they had read about in the preceding text, or incongruent. In the incongruent condition, one element of the scene was replaced by an element that was not mentioned in the text (for examples see Figure 1).

To verify or potentially expand the information about participants ability to retain the content information, we added the corresponding condition also in the subsequent *sentence reading task*. Thus, we included sentences that were exact repetitions of sentences in the preceding text. They differed with respect to the intervening picture task. In the congruent condition, the picture between the two identical presentations of the sentence (once in the text and once in the sentence task) fully corresponded to the sentence meaning. In the incongruent condition, the intervening picture did not fully correspond to the sentence meaning (i.e., depicting a deviant element). Any difference in reading times in the sentence task for the congruent versus incongruent conditions can thus be attributed to the influence of the congruent versus incongruent text-picture relations. Including a content information test in the sentence reading task also enables a direct statistical comparison of the retention effects of the content vs. surface linguistic form within one task (see *Joint Analysis of Content and Surface Linguistic Manipulations in the Sentence Reading Task*).

#### 4.1.1 Content information: picture task

**Figure 3:**
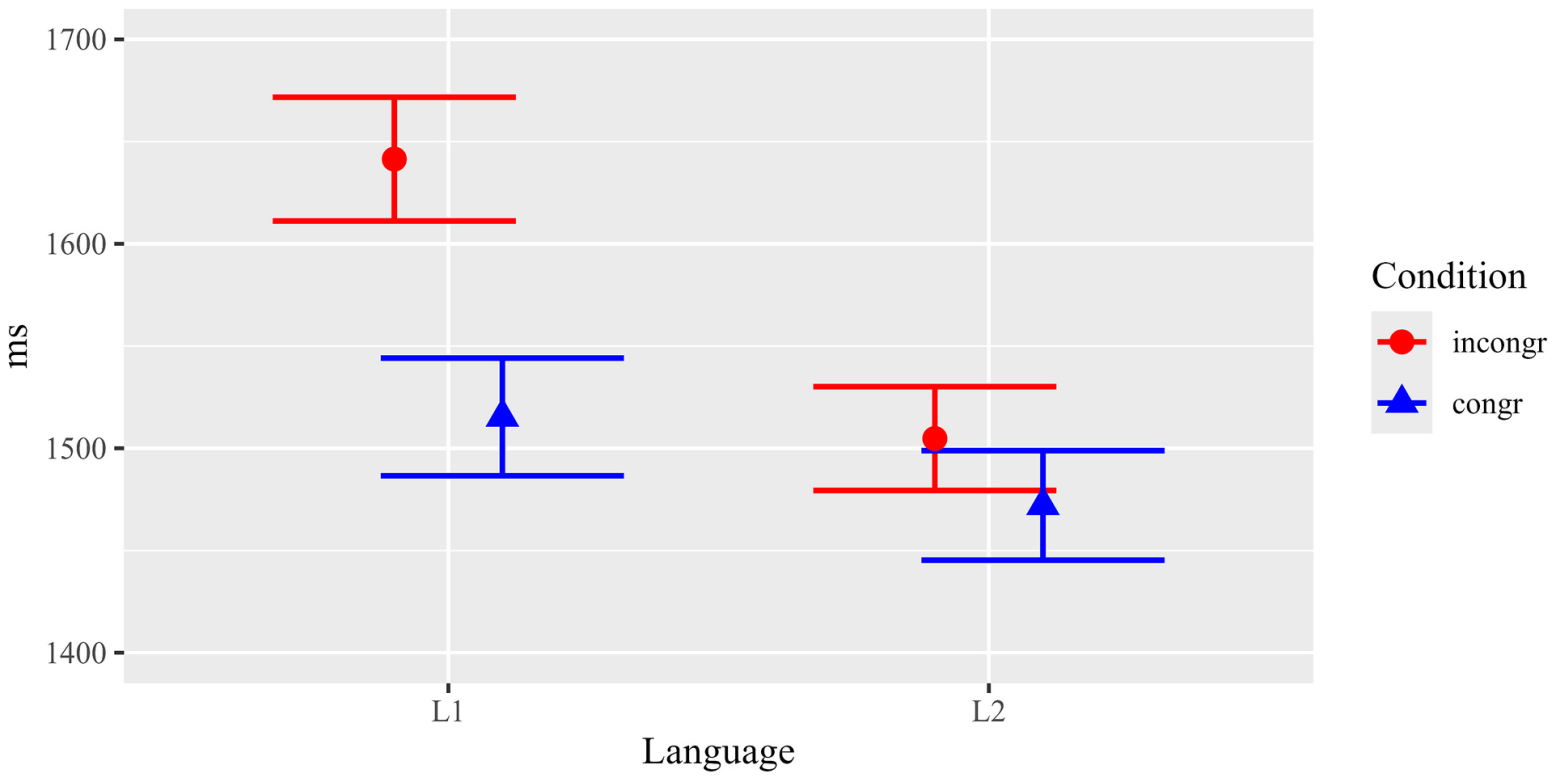
Results of the picture task, mean dwell times (with standard errors) in critical ROIs.

Mean dwell times (sum of all fixations) for the critical ROIs in the pictures (the congruent/incongruent element) are summarized in Figure 3 and Table 1A.

**Table 1:** Summary of results.

A: Content manipulation – Picture task						
	L1			L2		
	Mean	(SD)	*N*	Mean	(SD)	*N*
Incongruent	1,641.4	(959.9)	1,005	1,504.7	(805.1)	1,004
Congruent	1,515.3	(914.4)	1,009	1,472.0	(846.2)	998
Difference	126.1			32.7		

B: Content manipulation – Sentence task						
	L1			L2		
	Mean	(SD)	*N*	Mean	(SD)	*N*
Incongruent	785.2	(510.8)	1,024	1,041.2	(642.9)	1,022
Congruent	699.7	(484.8)	1,011	1,015.0	(657.7)	1,022
Difference	85.5			26.2		

C: Surface linguistic manipulation – Sentence task						
	L1			L2		
	Mean	(SD)	*N*	Mean	(SD)	*N*
Changed	1,795.6	(971.1)	1,023	2,604.4	(1,321.0)	1,023
Identical	1,766.1	(954.3)	1,024	2,464.8	(1,184.6)	1,022
Difference	29.5			139.6		

D: Number manipulation – Sentence task						
	L1			L2		
	Mean	(SD)	*N*	Mean	(SD)	*N*
Changed	895.1	(514.0)	505	1,327.9	(785.5)	506
Identical	852.2	(494.9)	506	1,255.9	(787.9)	506
Difference	43.9			72.0		

Mean of total durations (dwell times) per condition with standard deviations and number of observations.

Statistical analyses (see Table 2 for details) revealed a main effect of Condition (*F*(1, 61.4) = 10.81, *p* = 0.002) and a marginal trend for Language (*F*(1, 126.0) = 2.81, *p* = 0.096). Crucially, there was a significant interaction between both factors (*F*(1, 3765.5) = 4.46, *p* = 0.035).

**Table 2:** Mixed model ANOVA (type III) table for the results of the content manipulation in the picture task (dewll times in critical ROIs).

Fixed effects	Sum Sq	NumDF	DenDF	*F* value	Pr(>F)
Condition	4,488,419.1	1	61.4	10.81	0.002
Language	1,165,293.0	1	126.0	2.81	0.096
Condition: Language	1,852,349.7	1	3,765.5	4.46	0.035

Model structure: IA_DWELL_TIME ~ 1 + Condition *Language + (1 + Condition| picture_ID) + (1 | prtcpt_ID).

Results suggest that the effect of congruent versus incongruent text-picture relations was not the same in both populations. In order to resolve the interaction, subsequent separate mixed effects models were run for L1 and L2 participants. Results showed a significant effect of Content congruency for L1 (*F*(1, 1886.4) = 16.74, *p* < 0.001), but no significant difference for L2 (*F*(1, 1875.1) = 1.66, *p* = 0.197). This indicates that the main effect of Content congruency in the picture task was thus mainly driven by L1 participants. In sum, L1 participants showed significantly longer (126.1 ms) dwell times in critical regions when the depicted element was incongruent with the corresponding scene described in the preceding text (1641.4 ms) than when the depicted scene was congruent with the text (1515.3 ms). For L2 participants, the numerical difference between the two conditions was much smaller (32.7 ms) and not statistically significant.

The results imply that L1 participants encoded the meaning/content of the texts more strongly and possibly more extensively. Thus, they readily noticed deviations from the text content depicted in the pictures. In contrast, L2 readers did not manifest the same sensitivity to the content manipulation, indicating weaker and possibly less rich content representation of the text.

#### 4.1.2 Content information: sentence reading task

**Figure 4:**
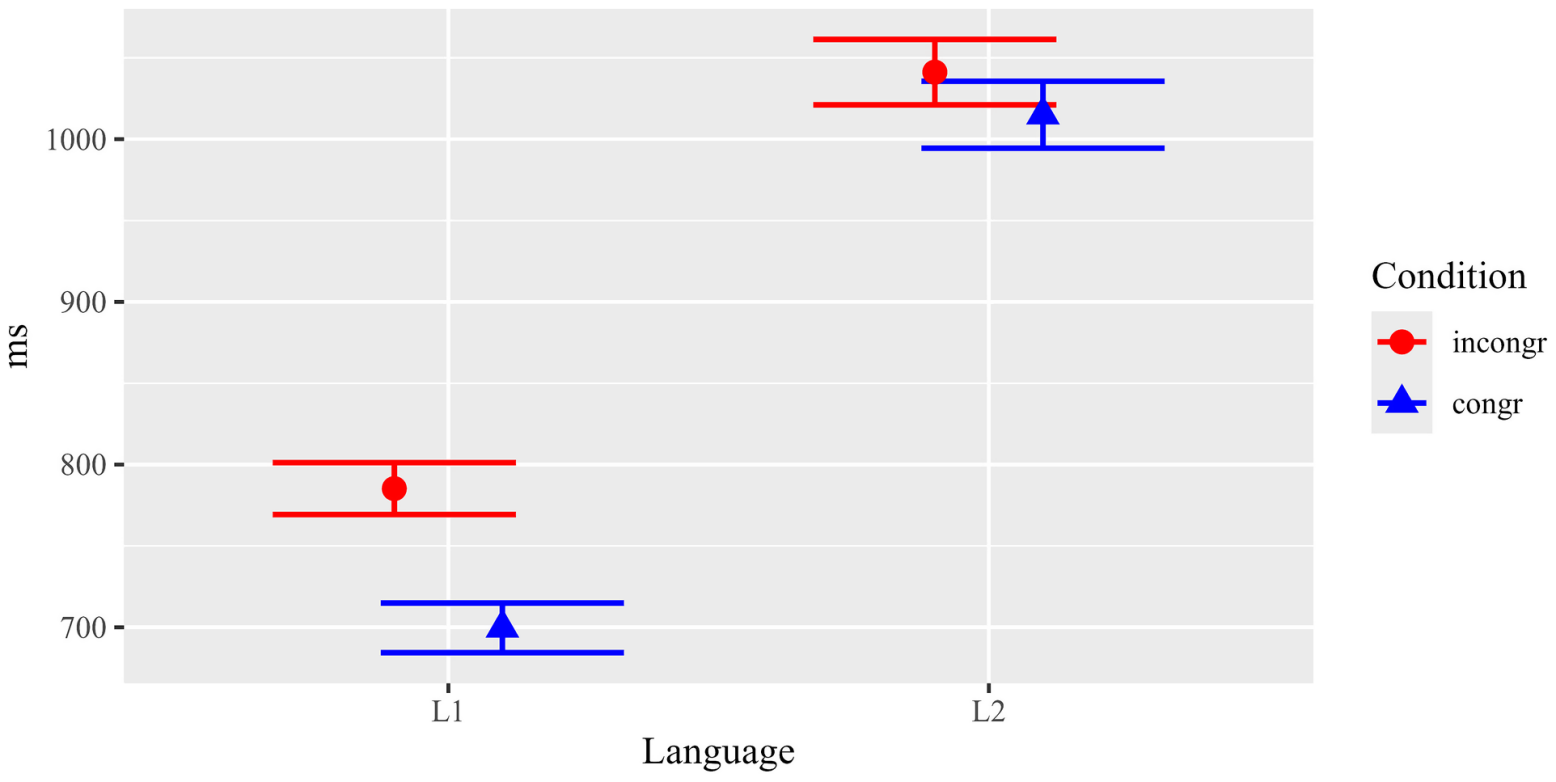
Results of the sentence reading task: Content manipulation. Mean total durations (with standard errors) in critical ROIs.

Results for reading times (total durations) of the critical region (critical noun phrase plus one word) are summarized in Figure 4 and Table 1B.

Statistical analyses (see Table 3) showed that there were main effects for Condition (*F*(1, 3887.8) = 15.65, *p* < 0.001) and Language (*F*(1, 113.6) = 31.62, *p* < 0.001) and, importantly, there was also a significant interaction between the two factors (*F*(1, 3887.8) = 4.61, *p* = 0.032).

**Table 3:** Mixed model ANOVA (type III) table for the results of the content manipulation in the sentence task (total durations in critical ROIs).

Fixed effects	Sum Sq	NumDF	DenDF	*F* value	Pr(>F)
Condition	3,157,736.1	1	3,887.8	15.65	<0.001
Language	6,379,752.7	1	113.6	31.62	<0.001
Condition: Language	929,147.4	1	3,887.8	4.61	0.032

Model structure: TOTAL_DURATION ~ 1 + Condition*Language + (1 | prtcpt_ID) + (1 + Language | sentence_pair_ID).

Separate analyses revealed that the effect of Condition was highly significant in L1 (*F*(1, 1939.5) = 23.8, *p* < 0.001), but there was no such effect in L2 (*F*(1, 1939.5) = 1.35, *p* = 0.246).

In parallel to the analysis of participants’ gazes at the pictures, the analysis of reading times of subsequent sentences confirmed that L1 participants showed a strong effect of content congruency between the information presented in the texts and the pictures, while for L2 participants no such effect was observed. Results of both tasks on the content manipulation thus indicate stronger or more robust/detailed content representations established during reading of the texts by L1 participants compared to L2 participants.

In addition to total durations for critical regions, we also analyzed early measures (gaze duration) and late measures (regression path duration). While the numerical trend for both measures was the same as for the total duration (more pronounced differences for L1 than for L2), the different effect of Content congruency for L1 versus L2 was statistically significant only for regressions, but not for gaze durations. This indicates that the robust effect found for total durations in L1 was mainly driven by regressions to the critical word.

### 4.2 Surface linguistic information

**Figure 5:**
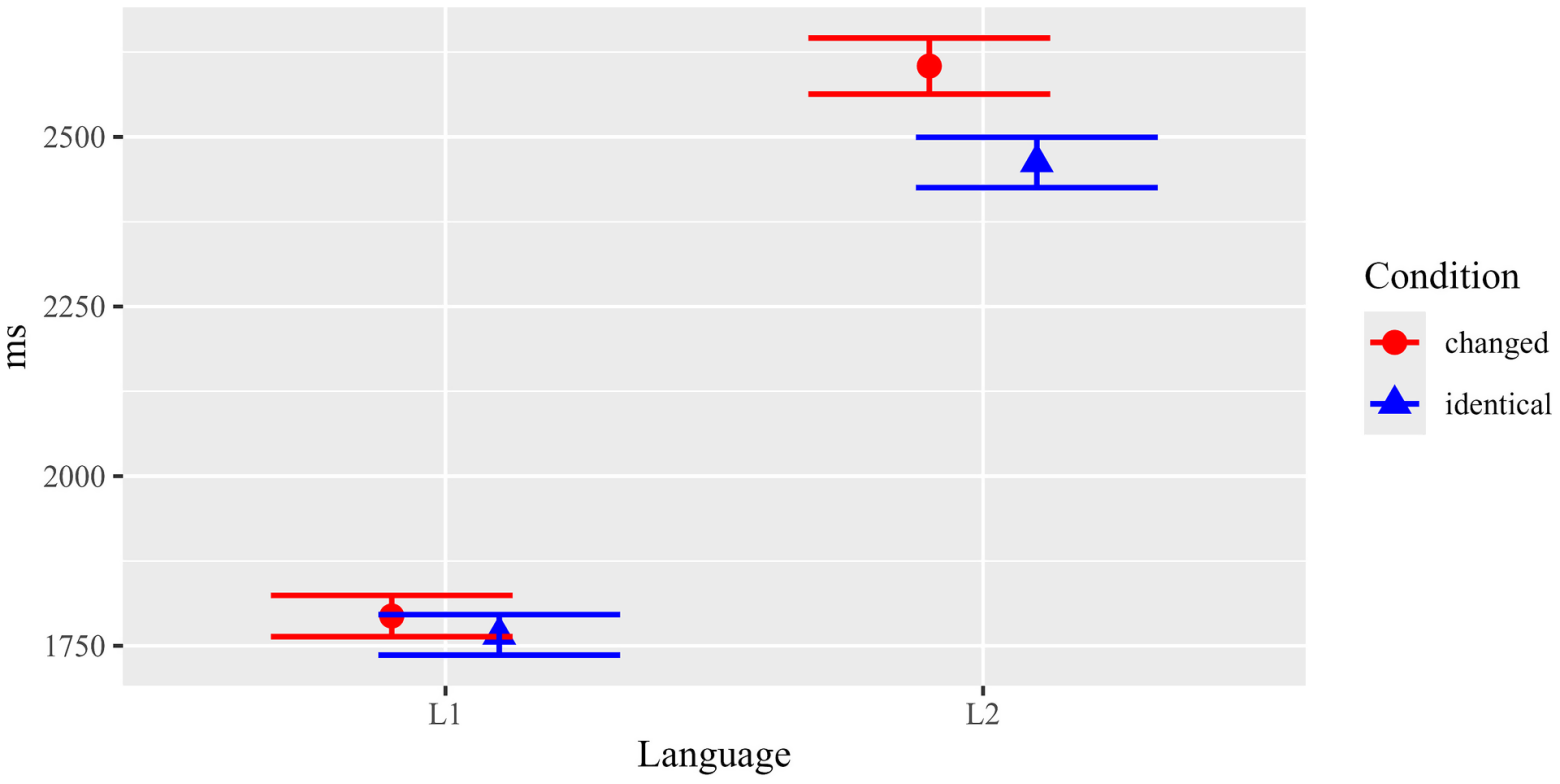
Results of the sentence reading task: Surface linguistic manipulation. Mean total durations (with standard errors) in critical ROIs.

Memory for surface linguistic information was tested in the sentence task by means of an identical/changed reading paradigm. It comprised a balanced number of two different grammatical alternations: grammatical voice (active vs. passive voice) and attribute position (attribute left vs. right of the noun). In the sentence reading task, the critical items were either identical with the ones presented in the preceding text phase of the experiment (e.g., active – active, left attribute – left attribute etc.), or their grammatical form was changed (e.g., passive – active, right – left etc.). Results for total fixation durations are summarized in Figure 5 and Table 1C. In general, total fixation durations were longer for the changed than for the identical conditions. This difference was larger in L2 (139.6 ms on average) than in L1 (29.5 ms on average).

Statistical analyses (see Table 4) confirmed the impression conveyed by the descriptive statistics: Beyond a main effect of Language (*F*(1, 142.5) = 46.85, *p* < 0.001), there was a main effect of Condition (*F*(1, 3902.0) = 12.62, *p* < 0.001), and – importantly – a significant interaction between the two factors (*F*(1, 3902.0) = 5.72, *p* = 0.017). Separate follow-up analyses for each of the two Language groups revealed that Condition was significant for L2 participants (*F*(1, 1950.0) = 13.33, *p* < 0.001), but not for L1 participants (*F*(1, 1952.0) = 1.00, *p* = 0.319). The results thus indicated that non-native participants were sensitive to the critical identical vs. changed manipulation, while no such indications were statistically observed for L1 participants.

**Table 4:** Mixed model ANOVA (type III) table for the results of the surface linguistic manipulation in the sentence task (total durations in critical ROIs).

Fixed effects	Sum Sq	NumDF	DenDF	*F* value	Pr(>F)
Condition	7,360,562.4	1	3,902.0	12.62	<0.001
Language	27,327,900.0	1	142.5	46.85	<0.001
Condition: Language	3,338,429.4	1	3,902.0	5.72	0.017

Model structure: TOTAL_DURATION ~ 1 + Condition*Language + (1 | prtcpt_ID) + (1 + Language | sentence_pair_ID).

Additional analyses were carried out in order to test whether the type of alternation (voice vs. attribute position) affected the general result that was observed averaged over both surface linguistic alternations. To this means, the factor “Alternation Type” (with “voice” and “attribute” as values) was added to the model.

The results showed a main effect of “Alternation Type” with shorter total fixations for the attribute manipulation than for the voice manipulation, most likely due to the ROI sizes (whole sentence for voice). However, there were no significant interactions between any of the other factors with “Alternation Type” and thus no indication that the observed interaction of “Condition: Language” would be fundamentally different between the two types of surface linguistic alternations tested, i.e., for voice vs. attribute position manipulation (see Table 5).

**Table 5:** Mixed model ANOVA (type III) table for the results of the surface linguistic manipulation in the sentence task by Alternation Type (voice vs. attribute).

Fixed effects	Sum Sq	NumDF	DenDF	*F* value	Pr(>F)
Condition	7,352,908.0	1	3,774.2	13.11	<0.001
Language	26,024,305.4	1	141.5	46.41	<0.001
AlternationType	35,741,774.3	1	32.7	63.73	<0.001
Condition: Language	3,333,274.1	1	3,774.2	5.94	0.015
Condition: AlternationType	137,660.6	1	3,774.2	0.25	0.620
Language: AlternationType	305.7	1	43.7	0.00	0.981
Condition: Language: AlternationType	430,029.3	1	3,774.2	0.77	0.381

Model structure: TOTAL_DURATION ~ 1 + Condition*Language*AlternationType + (1 + AlternationType | prtcpt_ID) + (1 + Language | sentence_pair_ID).

### 4.3 Joint analysis of content and surface linguistic manipulations in the sentence Reading task

A joint analysis was conducted to test whether the different patterns of results observed for the content and the surface linguistic information manipulations in the sentence reading task could be statistically substantiated. For this joint analysis, the two types of manipulation in the content versus surface linguistic information part of the experiment were joint under the factor Condition and coded with two corresponding levels: ‘unaltered’ (combining ‘congruent’ / ‘identical’), ‘manipulated’ (combining ‘incongruent’ / ‘changed’). Thus, in the unaltered condition, participants read sentences that were completely identical to the sentences in the text and for which they also had no incongruent input in the picture task. Sentences in the altered condition, in contrast, were either formally changed sentences (in the surface linguistic information manipulation) or participants were confronted with incongruent content information via the picture task (in the content manipulation). The mixed model for the joint analysis thus included Language (L1 vs. L2), Condition (unaltered vs. manipulated) and Type of Manipulation (surface linguistic information vs. content) as fixed effects. Results (see Table 6 for details) revealed a significant interaction of the three fixed effects Manipulation: Language: Type of Information (*F*(1, 790.8) = 9.70, *p* = 0.002). This interaction reveals that the effect of Manipulation (i.e., the core experimental manipulation of altered versus unaltered information in the follow-up sentence task) was influenced by Language and the type of information (at the surface linguistic level or the content level). The joint analysis therefore statistically substantiates the findings of the above reported different patterns for L1 and L2 participants in the surface linguistic versus content manipulation.

**Table 6:** Mixed model ANOVA (type III) table for the results of the joint analysis of the content manipulation and surface linguistic manipulation in the sentence task.

Fixed effects	Sum Sq	NumDF	DenDF	*F* value	Pr(>F)
Condition_2	9,971,479.6	1	7,790.8	25.38	<0.001
Language	19,451,324.8	1	157.8	49.51	<0.001
Manipulation	41,630,803.6	1	70.1	105.96	<0.001
Condition_2: Language	395,254.8	1	7,790.8	1.01	0.316
Condition_2: Manipulation	455,344.3	1	7,790.8	1.16	0.282
Language: Manipulation	11,300,756.2	1	143.0	28.76	<0.001
Condition_2: Language: Manipulation	3,811,130.0	1	7,790.8	9.70	0.002

Model structure: TOTAL_DURATION ~ 1 + Condition_2*Language*Manipulation + (1 + Manipulation | prtcpt_ID) + (1 + Language | sentence_pair_ID).

We thus conclude that our experimental manipulations result in different effects for the two populations depending on whether the content was manipulated, or whether surface linguistic information was manipulated. While L1 participants showed retention effects for content information, but not for surface linguistic information, for L2 participants the opposite pattern was observed: they showed retention effects for surface linguistic, but not for content manipulations.

**Figure 6:**
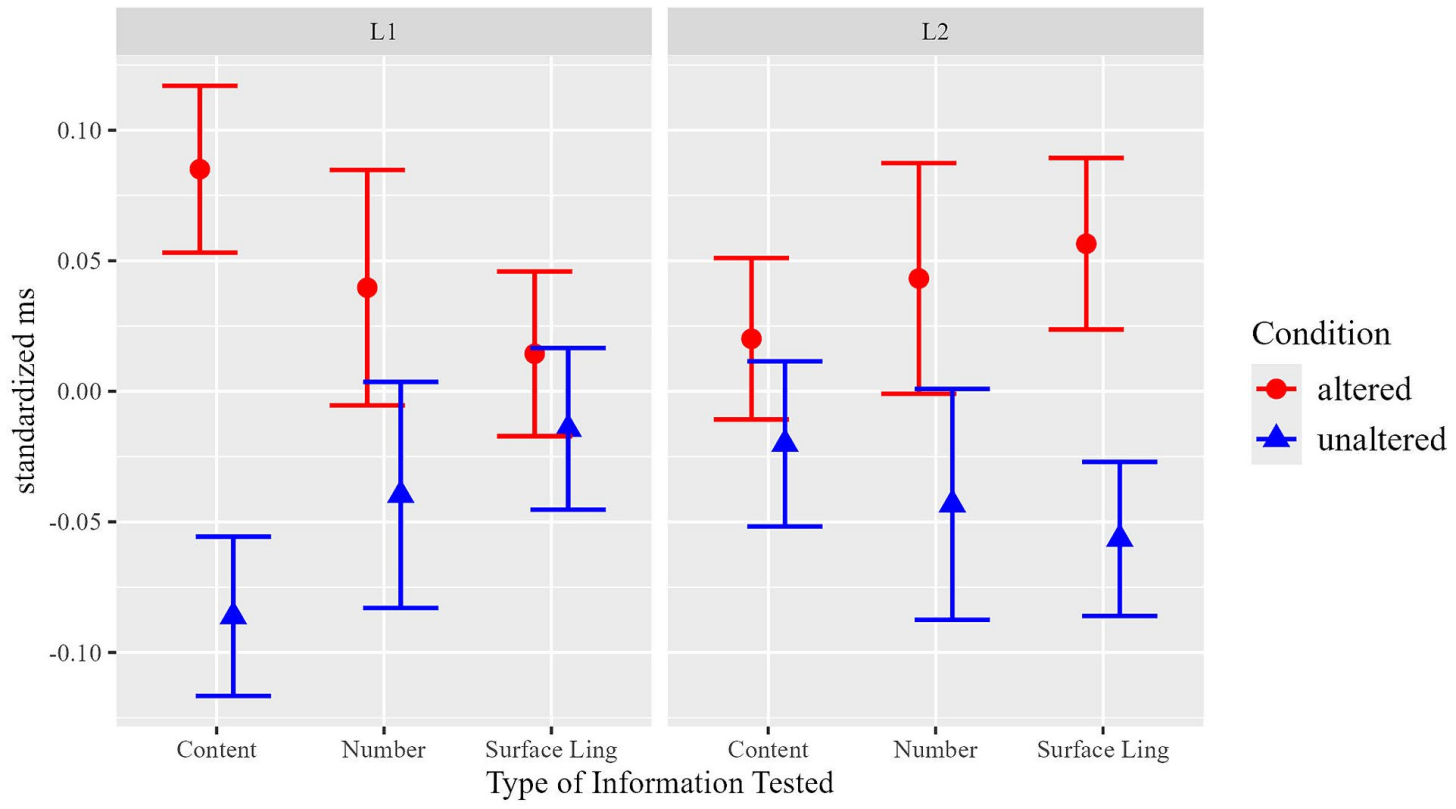
Summary of results of the sentence task. Means of total durations in critical ROIs, standardized within Language & Type of Manipulation.

### 4.4 Number information

**Figure 7:**
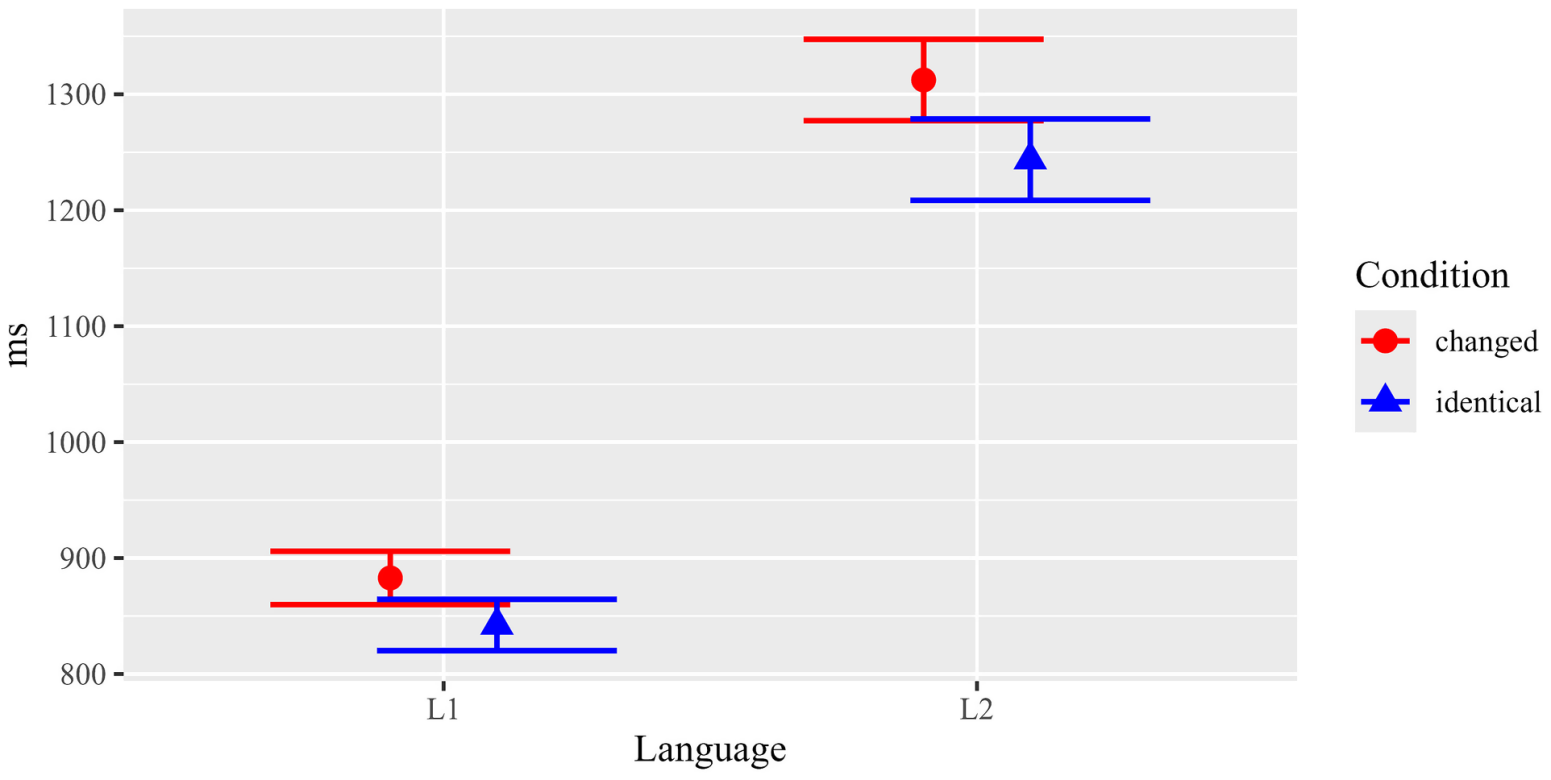
Results of the sentence reading task: Number manipulation. Mean total durations (with standard errors) in critical ROIs.

As mentioned above, we included a third type of manipulation in the sentence reading task for explorative insights. While the main focus of our experiment was a comparison of surface-level versus content information, we also included a smaller number of items for which we manipulated the number feature of nouns. Changes in number involve both a change in meaning/content as well as a formal change, in the case of plural morphology (a plural affix to the noun and inflectional changes on agreeing elements). Results for this manipulation (total durations) are summarized in Figure 7 and Table 1D.

Overall, there were longer total fixation durations for changed than for identical ROIs both in L1 (diff. 43.9 ms) and L2 (diff. 72.0 ms). Statistical analysis (see Table 7) revealed main effects of Language (*p* < 0.001) and Condition (*p* = 0.018), but no significant interaction of the fixed effects (*p* = 0.536). The main effect of Language shows generally slower reading times for L2 participants. Crucially, the main effect for Condition, suggest that, when averaged across both groups, participants were sensitive to the number manipulation, which included changes in both surface-level and content/meaning aspects.

**Table 7:** Mixed model ANOVA (type III) table for the results of the number manipulation in the sentence task (total durations in critical ROIs).

Fixed effects	Sum Sq	NumDF	DenDF	*F* value	Pr(>F)
Condition	1,548,139.1	1	1,887.0	5.56	0.018
Language	9,420,887.7	1	58.8	33.86	<0.001
Condition: Language	106,589.8	1	1,887.0	0.38	0.536

Model structure: TOTAL_DURATION ~ 1 + Condition*Language + (1 | prtcpt_ID) + (1 + Language | sentence_pair_ID).

### 4.5 Summary of results of the sentence reading task

In sum, our results show a contrasting pattern for content versus surface-level manipulations in both populations. While significant effects for content manipulation were observed only for L1 but not for L2 participants, the opposite pattern was found for surface-level manipulations, in which only L2 participants showed significant effects. For the additional number manipulation that combines changes in content and surface linguistic information, an effect of the experimental manipulation was observed across both populations.

Figure 6 summarizes all results of the sentence task. For this illustration, the factor Condition is represented with two levels: ‘unaltered’ (identical repetition, no manipulation) and ‘altered’ (i.e., ‘incongruent’ in the content manipulation, ‘changed’ in the form manipulation) In order to better illustrate the different effects of the factor Condition in our experimental manipulations (content, surface-level, and number), duration times are standardized (within manipulation type and language) in the figure to compensate for different length/durations between different manipulations and between L1 and L2.

## 5 General discussion

In the present study, we explored the retention of content and surface linguistic information in L1 and L2 German. In accordance with the initial hypotheses based on previous research, our results show that L1 and L2 readers store these two types of information in their mental text models to different extents. While L1 readers primarily retain information about the content, L2 readers show better retention of information about the surface linguistic information. The L1 results align with the previous studies on memory and mental text models (Anderson, 1974; Garnham and Oakhill, 1996; Johnson-Laird, 1977; Sachs, 1967, 1974): The surface linguistic information seems to decay from L1 readers’ memory soon after they establish the propositional meaning. In contrast, L2 readers were found to retain surface linguistic information in memory to a larger extent/longer than L1 readers, in accordance with recent studies (Bordag et al., 2021; Bordag and Opitz, 2025; Opitz et al., 2024; Sampaio and Konopka, 2013). In line with our findings on the retention of the content on the one hand and the voice and attribution information on the other is the observation that both L1 and L2 readers retain the information about number. The singular - plural alternation involves both propositional and surface linguistic changes. It is possible that the same outcome for both participant groups is a result of different underlying mechanisms: While the information retention in L1 might be primarily based on the memory for propositional meaning (one or several referents), the information retention in L2 might be primarily based on memory for the surface linguistic level (additional suffix and plural plus agreement changes within the critical number marked NP). Notably, as mentioned previously, all number manipulated NPs in the experiment were in the sentence subject position. As suggested by the results of Bordag and Opitz (2025), a syntactically prominent position might contribute to information retention by the L2 readers (see also Rogahn et al., 2018, on the role of prominent syntactic positions in incidental L2 acquisition).

The more extensive retention of surface linguistic information in L2 may be related to how L2 readers process language. Since their processing is less automatic, they need to allocate a greater proportion of cognitive resources to decoding linguistic forms (both at the word and morphosyntactic level) during reading. On the one hand, this may contribute to the retention of surface linguistic information, but on the other hand, it might leave reduced resources for the retention of content.

Even though the present results do not provide evidence that L2 readers would retain the explored pieces of content information, it indeed cannot be claimed that L2 readers do not retain content information at all. This is why we speak about the “extent” of retention, rather than about the ability to retain. The pieces of content information that we tested had various statuses in the texts participants read. The main criterion was that the tested information was well visualizable. Overall, the tested content information covered both central and peripheral information, in most cases they were however details that did not advance the main plot. Studies on the centrality effect and centrality deficit (in particular Miller and Keenan, 2011, but also Yeari et al., 2019; Yeari and Lantin, 2021) have shown that similarly to readers with attention deficit hyperactivity disorder (ADHD), L2 readers recall less information when reading in their L2 than when reading in their L1. However, the centrality deficit, i.e., recalling proportionally less central information than peripheral information, was observed only for less proficient L2 speakers. Proficient L2 readers, such as our participants, showed proportionally the same decrease in information recall for both central and peripheral information. It could therefore be the case that the amount of the tested information that the L2 readers retained was not sufficient to show significant effects. Numerically, though, we see a retention pattern for content information similar to L1 Future research testing larger proportions or different types of content information presented in the text might reveal an interaction in content retention between the two groups, with L2 participants showing a smaller but significant effect compared to L1 participants. Furthermore, future research could identify additional factors that contribute to information retention, in addition to syntactic prominence (i.e., the subject position of a piece of factual information). From a broader perspective, the observed differences in memory for content need to be replicated using different methodologies.

As outlined in the Introduction, the more extensive retention of surface linguistic information might be related to processing difficulties that L2 learners experience during reading. The SSH and the DP Model converge in predicting a dissociation between morphosyntactic processing and surface-form retention in adult L2 learners: while their online syntactic *processing* may remain shallow, their memory *representations* may preserve substantial surface-level detail. This theoretical convergence offers a compelling explanation for empirical findings showing robust retention of surface linguistic information in L2 comprehension, and suggests that the presence of such information in L2 mental text models might reflect a compensatory mechanism, whereby limited morphosyntactic parsing is offset by a stronger reliance on verbatim retention. This trade-off may allow L2 readers to maintain coherence and support comprehension despite reduced access to deeper grammatical structures.

Beyond its short-term benefits for comprehension, the retention of surface linguistic information may contribute to language acquisition through exemplar-based mechanisms. Within the usage-based framework, verbatim word sequences stored in memory facilitate the abstraction of linguistic regularities, contributing to mental grammar development through the gradual accumulation of distributional and semantic patterns. Thus, retention of surface linguistic information may also support the acquisition of grammatical properties expressed through these forms.

However, reduced memory for content in L2 readers can only be regarded as a disadvantage. It may hinder readers’ ability to construct coherent and integrated mental representations of texts, as well as to retain, recall, and build upon essential information across extended discourse. Such difficulties may impact academic performance and communication in professional and educational settings, suggesting that further research in this area could be valuable.

At least three limitations of our study should be noted, which also open up possible directions for future research. First, the L2 participant group was relatively homogeneous, which, while necessary for a controlled quantitative comparison with a matched L1 group, limits the generalizability of the findings. A significant proportion of the L2 participants had received formal German training in school and university settings in the Czech Republic, which may have contributed to their stronger focus on form over content, potentially reflecting the effect of teaching methods. Future research could examine whether different instructional approaches or acquisition contexts influence retention processes in L2 reading. An important aspect closely related to this is the role of individual metacognitive strategies. While the present study was not designed to investigate this issue, future research could benefit from triangulating behavioral data with retrospective self-reports, such as interviews. This approach could provide valuable insights into whether L2 learners systematically employ different reading strategies, particularly in balancing content comprehension and linguistic form. Second, all L2 participants were relatively advanced learners of German. Exploring retention of content and surface information across varying proficiency levels would provide further insights into the development of reading comprehension and clarify the mechanisms driving retention differences between content and form-based information. For example, to better understand the verbatim retention advantage in L2, it would be interesting to establish whether the surface-level retention effect is present in the early stages of L2 acquisition or emerges later in the process. Alternatively, one might hypothesize that surface-level retention is most prominent at lower proficiency levels, and that the relative weighting of different information types in memory approaches—and potentially even attains—native-like status at the most advanced proficiency levels. Third, the tested groups of participants of our study (L1 & L2) were chosen from two closely matched populations with respect to age, formal education. We focused on differences in retention between the native and non-native group. However, it is reasonable to assume that individual differences, for instance in working memory, may also contribute to the retention of different types of information. While we have no reason to assume that such individual aspects as working memory capacity systematically affected the observed crucial L1-L2 difference in our study, future research could clarify the relevance of individual cognitive characteristics such as working memory capacity or even metacognitive strategies for the retention of different types of information in reading.

In summary, our study provides evidence of differential memory effects in L1 and L2 reading. The findings suggest that while L1 readers outperform L2 readers in the retention of content information, L2 readers exhibit stronger retention of surface linguistic details. Further investigation of these differences could enhance our understanding of native and non-native disparities in educational success and outcomes, with potential implications for language learning and instructional approaches.

## Data Availability

The datasets, materials, and analyses scripts presented in this study can be found in online repositories: Open Science Framework (OSF) https://osf.io/b6dr4/.

## Footnotes

^1^Feminine nouns were excluded, as the definite feminine singular article is identical to the definite plural article *die* and therefore lower in salience than the masculine and neuter articles *der* and *das*.

^2^The first step applies thresholds for durations of 80 ms and a 0.5° distance, the second and third step have threshold durations of 50 ms and 140 ms, respectively, and a distance threshold of 1.25°. The fourth step sets minimum durations to 140 ms and maximum durations to 800 ms.

^3^Total fixation durations served as the most informative measure due to our experimental design and the need to include the whole clause into the ROI (for the voice alternation). Also, it has been demonstrated that total fixation durations (or total reading times) is one of the most robust measures for processes related to memory and/or expectation in reading (Conklin and Pellicer-Sánchez 2016; Pokhoday et al., 2023).
